# Epidemiological, clinical, and virologic features of two family clusters of avian influenza A (H7N9) virus infections in Southeast China

**DOI:** 10.1038/s41598-017-01761-w

**Published:** 2017-05-04

**Authors:** Jianfeng Xie, Yuwei Weng, Jianming Ou, Lin Zhao, Yanhua Zhang, Jinzhang Wang, Wei Chen, Meng Huang, Wenqiong Xiu, Hongbin Chen, Yongjun Zhang, Binshan Wu, Wenxiang He, Ying Zhu, Libin You, Zhimiao Huang, Canming Zhang, Longtao Hong, Wei Wang, Kuicheng Zheng

**Affiliations:** 1 Fujian Provincial Center for Disease Control and Prevention, Fujian Provincial Key Laboratory for Zoonoses Research, Fuzhou, 350001 Fujian Province China; 2 School of Public Health, Fujian Medical University, Fuzhou, 350004 Fujian Province China; 3 Key Laboratory of National Health and Family Planning Commission on Parasitic Disease Control and Prevention, Jiangsu Provincial Key Laboratory on Parasites and Vector Control Technology, Jiangsu Institute of Parasitic Diseases, Wuxi, 214064 Jiangsu Province China

## Abstract

This study aimed to investigate the epidemiological, clinical, and virologic characteristics of avian influenza A (H7N9) confirmed cases from two family clusters in Southeast China. Epidemiological data of the H7N9 confirmed cases and their close contacts were obtained through interviews and reviews of medical records. Of the four patients in these two family clusters, two cases had mild symptoms, one had severe symptoms, and one died. Three of the four patients had a history of exposure to live poultry or contaminated environments. The complete genome sequences of the H7N9 viruses from the same family cluster were highly homologous, and the four isolated viruses from the two family clusters exhibited the virologic features of the H7N9 virus, in terms of transmissibility, pathogenicity, host adaptation, and antiviral drug resistance. In addition, our findings indicated that the A/Fujian/18/2015 viral strain contained an additional hemagglutinin G225D substitution, which preferentially binds α2,6-linked sialic acids. The results of this study demonstrate that one family cluster was infected through common exposure to live poultry or contaminated environments, and the other was more likely to be infected through the human-to-human route.

## Introduction

The first confirmed case of avian influenza A (H7N9) virus infection was detected in Eastern China on March, 2013, and the infection spread rapidly across the eastern provinces of China, causing worldwide concern^1–3^. By July 19, 2016, 793 laboratory-confirmed H7N9 cases had been reported, including at least 319 deaths^4^. Most cases were detected in mainland China, and additional sporadic imported cases reported in Hong Kong, Taiwan, Malaysia, and Canada were attributed to travel to mainland China^5–8^. Since the first confirmed case of H7N9 infection was reported in Fujian Province, Southeast China, on April, 2013, there have been four epidemic waves of human H7N9 infections from 2013 through 2016, and 74 cases were detected by the end of August 2016, including two family clusters.

Most of the cases of human infection with this avian H7N9 virus were found to have recent exposure to live poultry or potentially contaminated environments, especially markets where live birds had been sold^9^. Several cases of family clusters have been reported since the outbreak of the novel H7N9 avian influenza virus in 2013 in China, with 8% of H7N9 infections occurring in clusters^10^, and most cases of the family clusters were found to share a common history of poultry exposure, resulting in difficulty in differentiating between common exposure and human-to-human transmission^1, 11^. Although healthcare workers with H7N9 infections have been reported in H7N9 clusters^12^, epidemiologic and virologic evidence demonstrates limited and non-sustainable human-to-human transmissibility of the H7N9 virus^13, 14^. On January 2015, two separate family clusters were identified in Fujian Province, Southeast China. In this study, we report the epidemiological, clinical, and virologic characteristics of four H7N9 cases from these two family clusters, and we investigated the possibility of human-to-human transmission in these clusters.

## Results

### Epidemiological profile of the two family clusters

During the period from 2013 through 2016, 294 suspected cases and laboratory-confirmed cases (including two cases that had contact with other H7N9-positive cases) of human infections with the H7N9 virus and two family clusters of H7N9 virus infection were identified in Fujian Province through the Chinese Surveillance System for Pneumonia of Unknown Origin^11^, and 676 close contacts were investigated through this system.

**Figure 1:**
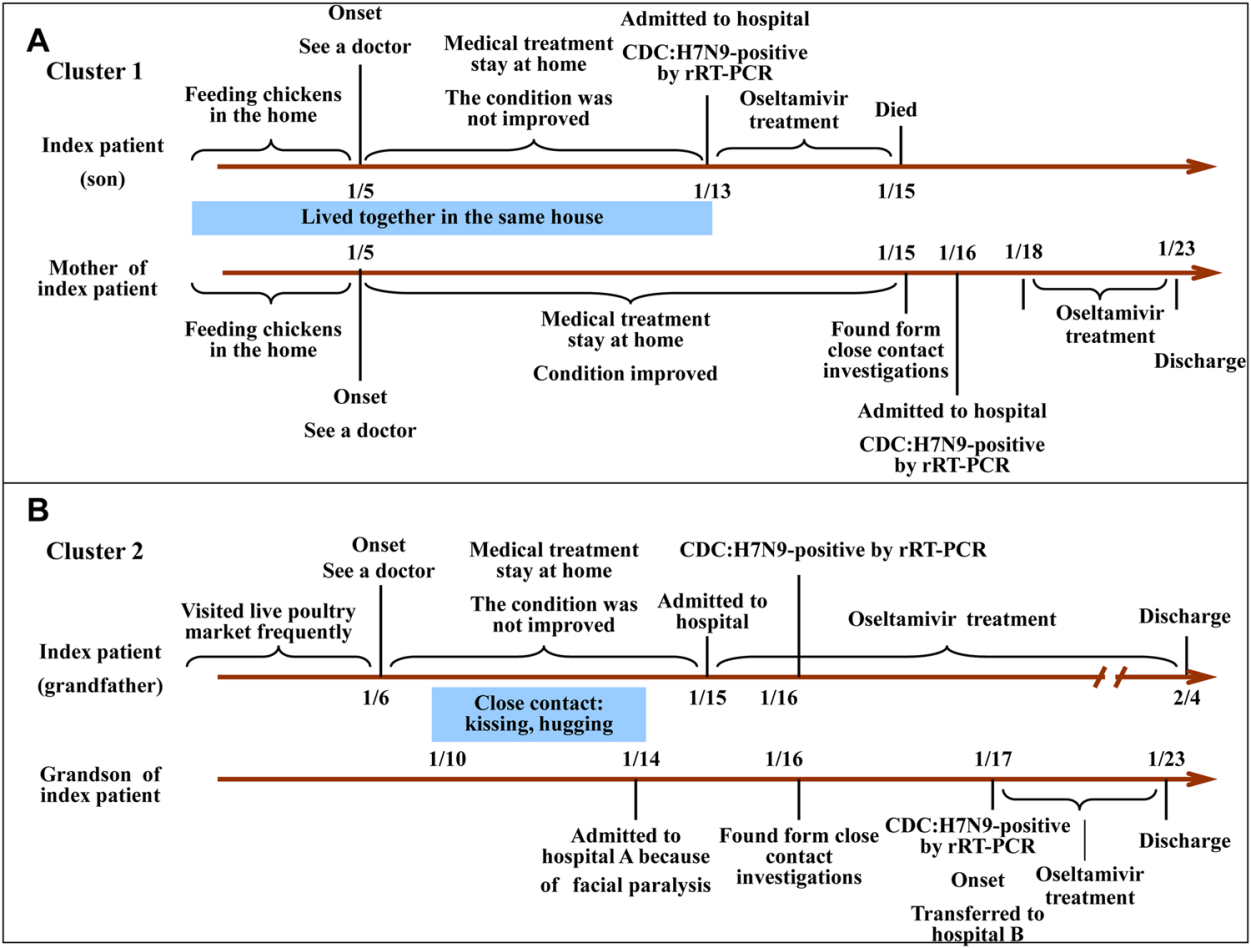
Time lines for two family clusters of H7N9 virus infection in Fujian, Southeast China.

The two family clusters had two household members each, and no medical workers were involved. All four patients were admitted to the hospital and received antiviral treatment with oseltamivir for several days, and one of them eventually died. No other contacts (five for cluster 1 and 10 for cluster 2), including eight family members and seven medical workers, developed influenza-like symptoms during the period of the investigation. Detailed epidemiological and clinical characteristics of the cases are described in Fig. 1 and Table 1.

**Table 1:** Demographic, epidemiological, and clinical characteristics for two family clusters of H7N9 virus infection in Fujian, China.

Characteristic	Cluster 1		Cluster 2	
	Index patient	Second patient	Index patient	Second patient
Age (yrs)/sex	54/M	77/F	58/M	2/M
Relation	Son	Mother	Grandfather	Grandson
Underlying medical disorders	No	Hypertension, glycuresis	No	No
Smoking (amount)	Yes (10 cigarettes/day)	No	Yes (20 cigarettes/day)	No
Exposure to live poultry or live poultry market within 7 days before illness onset	Yes	Yes	Yes	No
Date of illness onset	January 5, 2015	January 5, 2015	January 6, 2015	January 17, 2015
Signs of illness	Fever, chills, cough	Cough, dizziness	Fever, cough, weak	Fever
Date of admission	January 13, 2015	January 16, 2015	January 15, 2015	January 17, 2015
White cell count (×10^9^/liter)	2.92	9.70	2.76	10.42
Chest radiography	Bilateral pneumonia	Not applicable	Bilateral pneumonia	Not applicable
Oseltamivir treatment (duration of treatment)	January 13, 2015 (3 days)	January 18, 2015 (5 days)	January 15, 2015 (15 days)	January 17, 2015 (6 days)
Medication other than antivirals	Methylprednisolone, trachea cannula	No	Methylprednisolone, trachea cannula	No
Complications	ARDS, hypohepatia, renal insufficiency, cardiac failure, DIC	No	ARDS, hypohepatia	No
Outcome/date	Death, 01/15/2015	Recovery, 01/23/2015	Recovery, 02/04/2015	Recovery, 01/23/2015

The first family cluster comprised two persons with confirmed H7N9 infections (Fig. 1A). The index patient (the son) was a 54-year-old man with a fever of 40 °C at the onset of illness on January 5, 2014, and he saw a countryside doctor. However, his fever did not improve after 4 days of treatment. On January 13, the son was admitted to Putian Municipal No. 1 Hospital (Putian, China) because he experienced more severe coughing and shortness of breath. He was diagnosed with pneumonia of unknown origin and acute respiratory distress syndrome (ARDS), and the H7N9 virus was detected in his throat-swab samples by real-time reverse transcription polymerase chain reaction (RT-PCR). Oseltamivir was given as an antiviral treatment against H7N9 infection; however, the son, unfortunately, died on January 15. The second patient was the index patient’s 77-year-old mother, who was confirmed to have an H7N9 virus infection through monitoring and testing of close contacts with the confirmed index patient. On January 5, she saw a countryside doctor because of coughing and dizziness, and her physical condition improved following a 2-day treatment. She was investigated because of her close contact with her son, and her throat-swab specimen was collected and analyzed on January 15. On the following day (January 16), she was confirmed to have an H7N9 virus infection, and she was transferred to the Affiliated Hospital of Putian University (Putian, China) and placed in an isolated ward. Her vital signs and a routine blood test were normal during her stay in the hospital; however, oseltamivir was administered and she was not discharged from the hospital until two consecutive PCR assays of her throat-swab specimens were negative on January 22. The index patient lived with his mother in a two-floor house in Putian city, Fujian Province, where live poultry were freely bred in the backyard, which was full of chicken feathers and feces. According to the recollections of the patient’s family members, most of their neighbors bred live poultry, and some of the poultry died prior to his reported illness. Before the son became ill on January 5, his mother lived in extremely close contact with him, including feeding chickens, sitting, and eating together until January 13.

The second family cluster included a grandfather and his grandson with confirmed H7N9 virus infections (Fig. 1B) in Xiamen, Southeast China. The index patient (the grandfather) was a 58-year-old man that had a headache and chills with a normal body temperature at the onset of illness on January 6, 2015. At dusk, the grandfather visited a rural hospital and received an antondin injection and traditional Chinese medicine (no exact details are available). During the period from January 7 through 13, he had recurrent clinical manifestations, including weakness, coughing, shortness of breath, and fever, and his highest body temperature was 39.6 °C. Then, he visited Zhongshan Hospital of Xiamen University (Xiamen, China), and he was given cefotaxime treatment for 3 days. Because of the development of a more severe shortness of breath, the grandfather was hospitalized on January 15, and a routine blood test revealed a 2.75 × 10^9^/L white blood cell (WBC) count, which comprised 81% neutrophils and 16% lymphocytes. Oseltamivir, cefotaxime, doxycycline, and other symptomatic treatments were given for anti-infective therapy. He was diagnosed with pneumonia of unknown origin by consultation with multi-disciplinary experts on the morning of January 16, and his throat-swab specimens were collected and transferred to the local Center for Disease Control and Prevention to detect the H7N9 virus. On the afternoon of January 16, he was confirmed to have an H7N9 virus infection. Because his physical condition improved, he was discharged from the hospital on February 4. The second patient was the index patient’s 2-year-old grandson, who was admitted to Zhongshan Hospital because of facial paralysis. Because of his close contact with his grandfather, the grandson was investigated, and his throat-swab specimens were sampled on January 16. He was confirmed to have an H7N9 virus infection by RT-PCR on January 17, and he had a fever of 39 °C in the afternoon. Then, he was transferred to the First Affiliated Hospital of Xiamen University (Xiamen, China) and placed in an isolated ward. A routine blood test revealed a 1.04 × 10^10^/L WBC count, comprising 71% neutrophils and 13% lymphocytes. After the virus failed to be detected in the throat-swab specimen, the grandson was discharged from the hospital on January 23. The grandfather frequently visited a live poultry market near his house prior to the onset of his illness. After the grandfather became ill on January 6, he had close contact with his grandson (from January 10 to 14), including sitting and eating together, as well as kissing and hugging. The grandson did not visit any live poultry market, and he did not have any direct contact with live poultry.

### Phylogenetic characteristics of the genomes of viruses isolated from the two family clusters

**Figure 2:**
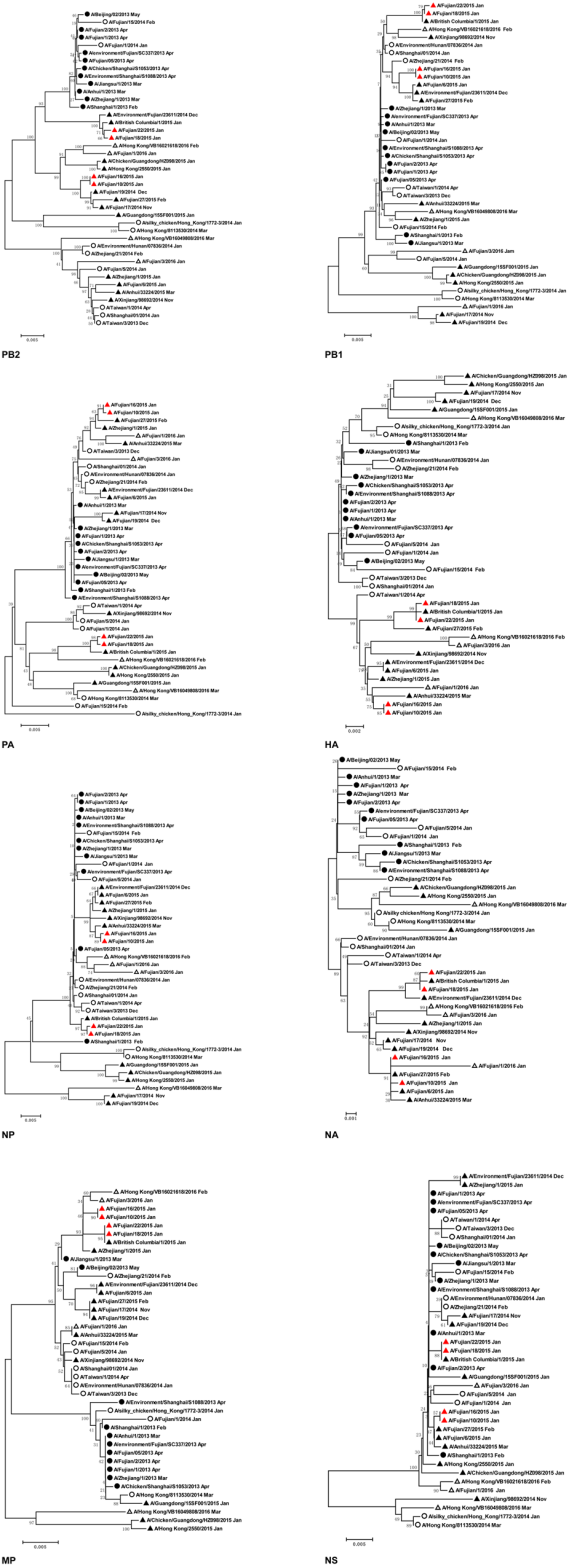
Phylogenetic tree of the complete genome of the H7N9 viruses isolated from two family clusters in Fujian province, Southeast China. H7N9 viruses isolated from the first, second, third, and forth epidemic waves of human infection with avian influenza A (H7N9) virus are marked with circles, open circles, triangles, and open triangles, respectively. The viruses isolated from the two family clusters are indicated by red triangles.

H7N9 viral strains were successfully isolated from the four patients, and all the virus genome sequences were obtained through DNA sequencing. To investigate the phylogenetic and genetic characteristics of the H7N9 viruses from these two family clusters, their complete genome sequences were analyzed and compared with several representative genome sequences available in the Global Initiative on Sharing Avian Influenza Data (GISAID) database (www.gisaid.org). Based on the temporal distribution of the H7N9 viral strains, 41 complete genome sequences of H7N9 viruses from the four epidemic waves were selected for phylogenetic analysis, and eight gene segments were closely related to each other, with ≥93.4% homology (Table 2). The viral strains isolated from the patients in the same family cluster shared high sequence similarities (Table 2), and all eight gene segments clustered together in the phylogenetic trees, with ≥99.9% (cluster 1) and ≥99.8% (cluster 2) identities in all eight gene segments at the nucleotide level. High sequence similarities were also observed between the complete genome sequences of the A/British Columbia/1/2015 viral strain from Canadian patients and the A/Fujian/18/2015 (the first patient from cluster 2) and A/Fujian/22/2015 viral strains from Xiamen, Fujian Province, China (Fig. 2), suggesting that two Canadian travel-associated cases became infected with the H7N9 virus in Xiamen. Table 2 and Fig. 2 show that the hemagglutinin (HA) and neuraminidase (NA) gene segments had fewer nucleotide substitutions during viral genetic evolution relative to the other six internal gene segments (those encoding polymerase basic protein 2 [PB2], polymerase basic protein 1 [PB1], polymerase acidic protein [PA], nucleoprotein [NP], matrix-2 protein [M2], and non-structural protein 1 [NS1]).

**Table 2:** The nucleotide identity of complete gene segments from selected representative H7N9 virus including the two family clusters.

Gene segments	All	Overall average	Cluster 1	Cluster 2
*PB2*	≥0.945	0.970	1	0.998
*PB1*	≥0.947	0.982	1	0.999
*PA*	≥0.952	0.980	1	0.999
*HA*	≥0.971	0.987	1	1
*NP*	≥0.934	0.979	0.999	0.999
*NA*	≥0.972	0.989	0.999	0.999
*MP*	≥0.961	0.983	1	1
*NS*	≥0.944	0.987	1	1

### Genetic characteristics of the genome of viruses from family clusters

Genetic characteristics of the four H7N9 viruses were analyzed from the two family clusters by comparing them with the genome sequence of a vaccine candidate strain (A/Anhui/1/2013). Forty-five amino acid variation sites were found among the four H7N9 viruses (Table 3). PB2 had the highest mutation frequency, with 13 amino acid substitutions, followed by the HA and NA proteins, each with nine substitutions. In addition, there were seven, three, two, one, and one amino acid substitutions in the PA, PB1, M2, NP, and NS1 proteins, respectively. Compared with the H7N9 vaccine candidate strain, the genomes of the A/Fujian/10/2015, A/Fujian/16/2015, A/Fujian/18/2015, and A/Fujian/22/2015 strains had mutations resulting in 20, 19, 32, and 30 amino acid substitutions, respectively, suggesting that the influenza A (H7N9) viruses had drifted from the vaccine candidate.

**Table 3:** Amino acid substitutions in H7N9 virus from the family clusters.

Gene	Amino acid position	H7N9 viral strains				
		A/Anhui/1/2013	A/Fujian/10/2015	A/Fujian/16/2015	A/Fujian/18/2015	A/Fujian/22/2015
*PB2*	106	T	A	A	—	—
	191	K	E	E	E	E
	199	A	—	—	S	S
	340	R	K	K	—	—
	453	P	—	—	H	H
	508	R	—	—	K	K
	534	S	—	—	F	—
	559	N	T	T	T	T
	588	A	V	V	—	—
	627	K	—	—	E	—
	701	D	—	—	N	N
	707	A	S	S	—	—
	711	N	—	—	S	S
*PB1*	209	K	N	N	—	—
	213	N	T	T	—	—
	398	D	N	N	—	—
*PA*	100	A	—	—	V	V
	262	R	—	—	K	K
	272	N	—	—	D	D
	343	A	—	—	S	S
	394	N	—	—	D	D
	419	D	—	—	G	G
	561	M	—	—	I	—
*HA*	46	N	—	—	S	S
	109	S	N	N	—	—
	134	A	V	V	—	—
	177	L	I	I	I	I
	225	G	—	—	D	—
	299	S	—	—	R	R
	387	E	A	A	A	A
	490	S	R	R	R	R
	497	M	—	—	I	I
*NP*	237	A	—	—	—	T
*NA*	16	I	T	T	T	T
	46	H	R	R	—	—
	114	V	—	—	I	I
	212	V	—	—	—	A
	245	S	P	P	P	P
	251	D	—	—	E	E
	308	V	—	—	I	I
	325	N	S	S	S	S
	405	G	V	—	—	—
*M2*	24	E	D	D	D	D
	27	V	—	—	I	I
*NS1*	206	S	—	—	C	C

Note. The *HA* gene was under the H3 numbering system and the *NA* gene under the N2 numbering system. Other internal genes were numbered from the start codon (M).

Because some key missense mutations in the genome of the H7N9 virus will alter viral virulence and host adaptation^15^, we examined the associations of the missense mutations in the H7N9 viruses from the two family clusters with mammalian adaptation, virulence, and antiviral drug resistance, and the results are described in Table 4. For the HA protein, all four H7N9 viruses contained G186V and Q226L substitutions (H3 numbering), which have been shown to increase the binding affinity of the H7N9 virus in the human upper respiratory tract^16, 17^, and the A/Fujian/18/2015 viral isolate contained an additional G225D substitution in the receptor binding site. For the NA protein, 118 R, 119E, 151D, 152R, 198N, 222I, 224R, 227E, 243D, 274H, 276E, 277E, 292R, 330D, 350 K, and 425E (N2 numbering) constitute its functional enzyme active sites, and mutations that alter these residues may cause reduced inhibition by NA inhibitors^18–20^. In our study, we did not find any drug resistance-associated substitutions in the NA protein. All the H7N9 viruses were resistant to amantadine and rimantadine because of the S31N mutation in the M2 protein. Amino acid changes, such as L89V, E627K, and D701N in PB2^21^, I368V in PB1^22^, N30D and T215A in the matrix-1 (M1) protein^23^, and P42S in NS1^24^, were reported to increase viral virulence or the transmission of avian influenza virus in mammals.

**Table 4:** Molecular analysis of H7N9 viruses from the family clusters.

Gene	Function	Mutation	A/Fujian/10/2015	A/Fujian/16/2015	A/Fujian/18/2015	A/Fujian/22/2015
*HA*	Virulence	Cleavage site	PEIPKR * GL	PEIPKR * GL	PEIPKR * GL	PEIPKR * GL
	Altered receptor	G186V	V	V	V	V
	specificity	G225D	G	G	D	G
		Q226L	L	L	L	L
*NA*	Antiviral resistance	E119V	E	E	E	E
	(Oseltamivir)	I222K/R	I	I	I	I
		R292K	R	R	R	R
	Increased virulence in mice	Stalk	69-73AA deletion	69-73AA deletion	69-73AA deletion	69-73AA deletion
*PB2*	Enhanced polymerase activity and increased virulence in mice	L89V	V	V	V	V
		E627K	K	K	E	K
	Enhanced transmission in guinea pigs	D701N	D	D	N	N
*PB1*	Increased transmission in ferret	I368V	V	V	V	V
*PB1-F2*	Increased pathogenicity in mice	87–90 amino acids in length	90 AA	90 AA	90 AA	90 AA
*PA*	Species-associated signature positions	V100A	A	A	V	V
		K356R	R	R	R	R
		S409N	N	N	N	N
*M1*	Increased virulence in mice	N30D	D	D	D	D
		T215A	A	A	A	A
*M2*	Antiviral resistance (Amantadine)	S31N	N	N	N	N
*NS1*	Increased virulence in mice	P42S	S	S	S	S

Note: The *HA* gene was under the H3 numbering system and the *NA* gene under the N2 numbering system. Other internal genes were numbered from the start codon (M).

In addition, all H7N9 viruses had five potential glycosylation sites (30NGT, 46NAT, 249NDT, 421NWT, and 493NNT) in the HA protein and seven potential glycosylation sites (42NCS, 52NTS, 63NET, 66NIT, 82NLT, 142NGT, and 197NAS) in the NA protein.

## Discussion

In early 2013, an outbreak of H7N9 virus infections occurred in Eastern China, which generated global concern^1^. To date, the virus has caused four outbreaks in humans, and most cases occurred during winter and spring^25^. The H7N9 virus is a low pathogenic avian influenza A virus, and it does not cause identifiable illness or death in poultry, whereas humans act as sentinels for the presence of these viruses in avian infections^17^. Currently, humans get infected with the H7N9 virus predominantly through contact with asymptomatic poultry, and sporadically via direct contact or exposure to environments that are contaminated with infected poultry^1^. However, there are increasing reports of clusters of human cases infected with the H7N9 virus, among which some cases have close contacts and some share common exposure to live poultry or live poultry markets, thereby resulting in difficulty in identifying viral transmission routes (either via common exposure or human-to-human transmission)^26, 27^. However, close, persistent, and unprotected contacts with symptomatic cases with H7N9 virus infections may cause human-to-human transmission, although this transmissibility is limited and non-sustainable^13, 14^. Therefore, it is important to examine the likelihood of efficient and sustained human-to-human transmission of the H7N9 virus. Here, we reported the epidemiological, clinical, and virologic characteristics of two family clusters with H7N9 virus infections in Southeast China to clarify the transmission and pathogenicity of the virus.

A phylogenetic analysis showed that all eight gene segments of H7N9 viruses in the same family cluster shared high sequence similarities and clustered together in a single branch of a phylogenetic tree, which suggests that the index patients and secondary patients from the family clusters were infected via human-to-human transmission or through exposure to common live poultry or contaminated environments. We collected specimens from live-poultry markets neighboring the patients’ houses, and H7N9 viruses were detected in the poultry washing water and cage surface cleaning wipes by RT-PCR (Supplementary Table S1); however, it was unfortunate that the H7 and N9 gene sequences were not obtained at that time. It was estimated previously that the mean incubation period of the H7N9 virus is 3.3 days (95% confidence interval [CI], 1.4 to 5.7 days), with a 0.76 coefficient of variation (95% CI, 0.1 to 5.5)^28, 29^. The onset of H7N9 infection-like syndromes occurred on the same day in the two patients from cluster 1 (January 5, 2015), and the time required for human-to-human transmission was not met, implying a simultaneous or common route of infection. For cluster 2, the index patient visited a live poultry market frequently prior to the onset of his illness (January 6, 2015), with close contact with the secondary patient from January 10 to 14. The secondary patient became ill on January 17 without exposure to any live poultry market or direct contact with any poultry. Therefore, we believe that the most likely route of infection was human-to-human transmission for cluster 2.

Gene mutations of the H7N9 virus have been demonstrated to change its pathogenicity, transmissibility, host adaption, and drug resistance^30–32^. Amino acid substitutions in the PB2 protein (L89V, K526R, Q591K, E627K, and D701N), PB1-F2 protein (N66S), M1 protein (N30D and T215A), and NS1 protein (P42S and D92E) increased H7N9 virulence in mice^33–35^, and amino acid substitutions in the HA protein (G186V and Q226L), PB2 protein (D256G and K702R), PA protein (V100A, K356R, and S409N), and NS1 protein (N205S and G210R) altered species-associated signature positions^36^. Previous studies have shown that the PB1 I368V mutation may increase the transmissibility of the H7N9 virus in ferrets^37^, and the PB1 L589P mutation and the PA L336M mutation increase H7N9 viral replication in mammalian cells^36^. In addition, the NA mutations E119V, I22K/R, and R292K resulted in a reduction in the sensitivity of the H7N9 virus to NA inhibitors^38^, and the M2 S31N mutation may cause the H7N9 virus to become resistant to adamantine-derived drugs^39^.

To analyze the virulence, transmissibility, drug-resistance, and antigenicity of the H7N9 virus from the family clusters, we performed an in-depth analysis of the genetic characteristics of the four H7N9 viruses. The four viral isolates from the two family clusters showed the features of the avian influenza A (H7N9) virus as reported previously^40^, in terms of their transmissibility, pathogenicity, host adaptation, and antiviral drug resistance. In addition, our data indicated that the A/Fujian/18/2015 virus contained an additional HA G225D substitution (the receptor binding site), which binds preferentially to α2,6-linked sialic acids and may increase the likelihood of viral upper respiratory tract transmission^41, 42^. It was reported that the HA G225D mutation in the H1N1 virus potentially contributes to the specificity of HA for the sialic acid receptor Sia(α2-6) Gal, thereby enabling the replication and transmission of the virus within and among humans^43^. In addition, we found that all four isolates were resistant to amantadine and rimantadine because of the S31N substitution in the M2 protein^44^. However, no substitutions were detected in the enzyme-active functional domains of the NA protein, suggesting that it is sensitive to oseltamivir and zanamivir. Thus, early clinical suspicions or confirmed cases of H7N9 virus infection and early administration of oseltamivir may help to reduce the severity of the disease. Since 2013, the HA and NA proteins of the H7N9 virus have maintained stable potential glycosylation sites, indicating that glycosylation may not be the factor affecting viral evolution and virulence.

Our study has several limitations. First, the epidemiological data were collected primarily from the patients’ family members, which may cause a potential recall bias. Second, detailed exposure information is unavailable, such as the duration, frequency, and intensity of the exposure. Third, we detected the H7N9 virus in the poultry washing water and cage surface cleaning wipes sampled from the live-poultry markets that were close to the patients’ houses; however, the H7 and N9 gene sequences were not obtained, and, therefore, they were not available for the phylogenetic analysis of the H7N9 virus. Finally, paired serum samples should have been collected during the acute and convalescent stages of the patients and their contacts for further assessment of the potential for secondary human-to-human H7N9 virus transmission, including the potential identification of asymptomatic infections.

In summary, the results of this study demonstrate that one family cluster was infected with H7N9 through exposure to the same live poultry or contaminated environments, and the other was more likely to be transmitted through the human-to-human route. Until now, there has been no stable and effective human-to-human transmission of H7N9 viruses; however, increasing evidence shows that the H7N9 virus has become better adapted to mammalian infections^3, 45, 46^. Therefore, it is of great importance to strengthen the surveillance of the H7N9 virus among poultry and humans, confirm the source of infections, trace and investigate close contacts with confirmed cases, complete viral isolation from H7N9 patients quickly, and emphasize individual protective interventions. In addition, virologic analyses to assess genetic changes of the H7N9 virus are of great value to determine the viral transmissibility among humans, as well as the pandemic potential.

## Methods

### Ethical considerations

This study was approved by the Ethical Review Committee of the Fujian Provincial Center for Disease Control and Prevention (FJCDC-20150001). Signed inform consent was obtained from all participants following a detailed description of the purpose of the study, and all participants agreed to publish related demographic and clinical features. This study was performed in accordance with national and local laws and guidelines.

### Study subjects

Suspected cases of H7N9 virus infection among hospitalized patients with pneumonia were identified through the “Field investigation” component in the Chinese Surveillance System for Pneumonia of Unknown Origin, which was established in 2004^47^. Two-week enhanced surveillance was implemented in the regions where confirmed H7N9 cases were diagnosed, in which patients with influenza-like illness or severe acute respiratory infection admitted to secondary and higher health institutions were assessed for an H7N9 virus infection^4^. Once an H7N9 infection was confirmed, those with close contacts with the confirmed cases were traced and an active investigation was performed. The procedures for determining and managing suspected cases, confirmed cases, and close contacts were performed according to national guidelines^48, 49^.

### Epidemiological investigation and sample collection

Once patients were identified as suspected cases of H7N9 virus infection, epidemiological data of the suspected cases and their close contacts (including patients’ family members, neighbors, healthcare workers, and live poultry dealers in the live-poultry markets) were obtained through interviews with the patients and their family members, as well as reviews of the medical records. Possible exposure history, clinical characteristics, and medical care for suspected cases were collected. Respiratory specimens were collected from suspected cases and their contacts who had influenza-like symptoms, and they were transferred rapidly to the local Center for Disease Control and Prevention in a cryogenic storage container.

### Case confirmations

Respiratory specimens were processed according to the Chinese Guideline of Diagnosis and Treatment for Human Infections with the Avian Influenza A (H7N9) Virus issued by the National Health and Family Planning Commission of China (NHFPCC)^49^.

### Virus isolation

To isolate the H7N9 virus, 0.2-ml throat-swab specimens of confirmed cases were injected into the allantoic sac of 9-to-10-day-old specific pathogen-free embryonated chicken eggs and incubated at 35 °C for 3 days in a biosafety level 3 laboratory. The titer of the virus was identified by hemagglutination assays, and hemagglutination-positive allantoic fluids were collected. The H7N9 virus was detected with an RT-PCR assay using a protocol described previously^50^.

### Genome sequencing and phylogenetic analysis

RNA was extracted from allantoic fluids with the QIAamp Viral RNA Mini Kit (Qiagen GmbH, Hilden, Germany) following the manufacturer’s instructions. An RT-PCR analysis was performed with the OneStep RT-PCR kit (Qiagen GmbH) using 22 pairs of primers (Supplementary Table S2) under the following conditions: 60 °C for 1 min, 42 °C for 10 min, 50 °C for 30 min, 95 °C for 15 min, followed by 35 cycles of 94 °C for 30 s, 55 °C for 30 s, and 72 °C for 90 s, and finally at 72 °C for 10 min. PCR products were purified from 1% agarose gels using the QIAquick Gel Extraction Kit (Qiagen GmbH)^51^^.^

Nucleotide sequencing was performed on an ABI 3500 genetic analyzer (Life Technologies, Grand Island, NY, USA) using the ABI BigDye Terminator v3.1 Cycle Sequencing Kit (Life Technologies), following the manufacturer’s instructions. The sequencing primers were M13F (5′–TGTAAAACGACGGCCAGT–3′) and M13R (5′–CAGGAAACAGCTATG ACC–3′). Sequences were assembled using the SeqMan program of the Lasergene Package (DNASTAR, Inc.; Madison, WI, USA). Nucleotide alignments and a phylogenetic tree were constructed using MEGA version 5.1 (The Biodesign Institute, Tempe, AZ, USA) by the neighbor-Joining method with 1,000 bootstrap replicates.

Full genome sequences of the viruses from these patients were submitted to the GISAID database (http://platform.gisaid.org) on June 15, 2016. To construct phylogenetic trees for each gene segment, the available full-length sequences of selected influenza A (H7N9) viruses were downloaded from GISAID databases and used as references (accession numbers are provided in Supplementary Table S3).

## Electronic supplementary material

Supplementary Tables
