# Supplementary material for: Epidemiological, clinical, and virologic features of two family clusters of avian influenza A (H7N9) virus infections in Southeast China
Source: Sci Rep. 2017 May 4;7:1512. doi: 10.1038/s41598-017-01761-w (PMC5431426; doi:10.1038/s41598-017-01761-w)
Supplement: Supplementary file 1 — Supplementary Tables [file 41598_2017_1761_MOESM1_ESM.pdf]

Epidemiological, clinical and virologic features of two family clusters of avian influenza A (H7N9) virus infections in Southeast China

Jianfeng Xie[1,2※], Yuwei Weng[1,2※], Jianming Ou[1,2,※], Lin Zhao[1], Yanhua Zhang[1], Jinzhang Wang[1], Wei Chen[1], Meng Huang[1], Wenqiong Xiu[1], Hongbin Chen[1], Yongjun Zhang[1], Binshan Wu[1], Wenxiang He[1], Ying Zhu[1], Libin You[1], Zhimiao Huang[1], Canming Zhang[1], Longtao Hong[1,2], Wei Wang[2,3*], Kuicheng Zheng[1,2*]

[1] Fujian Provincial Center for Disease Control and Prevention, Fujian Provincial Key Laboratory for Zoonoses Research, Fuzhou 350001, Fujian Province, China;

[2] School of Public Health, Fujian Medical University, Fuzhou 350004, Fujian Province, China;

[3] Key Laboratory of National Health and Family Planning Commission on Parasitic Disease Control and Prevention, Jiangsu Provincial Key Laboratory on Parasites and Vector Control Technology, Jiangsu Institute of Parasitic Diseases, Wuxi 214064, Jiangsu Province, China.

※ These authors contributed equally to this work.

* Corresponding authors. Dr. Wei Wang, Key Laboratory of National Health and Family Planning Commission on Parasitic Disease Control and Prevention, Jiangsu Provincial Key Laboratory on Parasites and Vector Control Technology, Jiangsu Institute of Parasitic Diseases, 117 Yangxiang, Meiyuan, Wuxi City, Jiangsu Province, 214064, China, E-mail: wangwei@jipd.com; or Dr. Kuicheng Zheng, Fujian Provincial Center for Disease Control and Prevention, Fujian Provincial Key Laboratory for Zoonoses Research, No. 76 Jingtai Road, Fuzhou City, Fujian Province, 350001, China, E-mail: kingdadi9909@126.com.

Table S1 Detection of influenza A (H7N9) virus in the specimens from patients' neighboring live-poultry markets

Types of specimens	Cluster 1 Hushi Market	Cluster 2 Xiwan Market
Poultry cage surface wipes	(1/1)	(0/7)
Poultry washing water	(2/2)	(1/10)
Placing poultry board surface wipes	(0/1)	(0/3)

Table S2 Primers for amplification of H7N9 avian influenza virus used in RT-PCR

Primers	Sequence (5'to3')	Fragments＆ Length
PB2-F0	TGTAAAACGACGGCCAGTAGCGAAAGCAGGTC	PB2-1 (900+36)
PB2-R870	CAGGAAACAGCTATGACCTCTCTGTACTATGGC	
PB2-F620	TGTAAAACGACGGCCAGTTGGAGAGAGAACTGGTTC	PB2-2 (1192+36)
PB2-R1811	CAGGAAACAGCTATGACCCGCATCTGYTGGAATAG	
PB2-F1619	TGTAAAACGACGGCCAGTATGGTCCGGAATCAGTG	PB2-3 (723+36)
PB2-R2341	CAGGAAACAGCTATGACCAGTAGAAACAAGGTCGTTT	
PB1-F0	TGTAAAACGACGGCCAGTAGCGAAAGCAGGCA	PB1-1 (740+36)
PB1-R700	CAGGAAACAGCTATGACCCTCTTTCAGCATC	
PB1-F604	TGTAAAACGACGGCCAGTCAGAGAACAATAG	PB1-2 (1181+36)
PB1-R1784	CAGGAAACAGCTATGACCCCTCCATCTGAAACCAA	
PB1-F1569	TGTAAAACGACGGCCAGTGAGCRTTGGTGTTAC	PB1-3 (772+36)
PB1-R2340	CAGGAAACAGCTATGACCAGTAGAAACAAGGCATTT	
PA-F0	TGTAAAACGACGGCCAGTAGCGAAAGCAGGTAC	PA-1 (767+36)
PA-R730	CAGGAAACAGCTATGACCCCTCAATGCAGCC	
PA-F550	TGTAAAACGACGGCCAGTAGCAGGGGTCTAT	PA-2 (1212+36)
PA-R1761	CAGGAAACAGCTATGACCTTGRAGAAGGCAGCG	
PA-F1557	TGTAAAACGACGGCCAGTCTTTGTAAGTATGG	PA-3 (677+36)
PA-R2233	CAGGAAACAGCTATGACCAGTAGAAACAAGGTACTT	
HA-F0	TGTAAAACGACGGCCAGTAGCAAAAGCAGGGG	HA-1 (784+36)
HA-R754	CAGGAAACAGCTATGACCACTGTATCATTGGG	
HA-F633	TGTAAAACGACGGCCAGTGACAGTTGGGAGTTC	HA-2 (1089+36)
HA-R1721	CAGGAAACAGCTATGACCAGTAGAAACAAGGGTGTTTT	
HA-F1091	TGTAAAACGACGGCCAGTGACACCAGAATGCAC	HA-3 (631+36)
HA-R1721	CAGGAAACAGCTATGACCAGTAGAAACAAGGGTGTTTT	
NP-F0	TGTAAAACGACGGCCAGTAGCAAAAGCAGGGTA	NP-1 (673+36)
NP-R619	CAGGAAACAGCTATGACCAGAAATTCCGGTC	
NP-F387	TGTAAAACGACGGCCAGTAACTGCTGGTCTTAC	NP-2 (991+36)
NP-R1377	CAGGAAACAGCTATGACCCTGGAATGACAC	
NP-F988	TGTAAAACGACGGCCAGTTGGATGGCATGCCAC	NP-3 (578+36)
NP-R1565	CAGGAAACAGCTATGACCAGTAGAAACAAGGGTATTTTT	
NA-F1	TGTAAAACGACGGCCAGTAGCAAAAGCAGGAGTGAAAA	NA-1 (496+36)
NA-R480	CAGGAAACAGCTATGACCTGATAGTGGCCAGC	
NA-F257	TGTAAAACGACGGCCAGTGGCTCTGTACTATAAATTC	NA-2 (1030+36)
NA-R1286	CAGGAAACAGCTATGACCTTGGGTCTTCCACG	

NA-F902	TGTAAAACGACGGCCAGTAGATAGACCCAGTAGC	NA-3	(530+36)
NA-R1431	CAGGAAACAGCTATGACCAGTAGAAACAAGGAGTTTTTT		
M-F0	TGTAAAACGACGGCCAGTAGCAAAAGCAGGTAG	M-1	(655+38)
M-R638	CAGGAAACAGCTATGACCACCATTTGCCTAGC		
M-F365	TGTAAAACGACGGCCAGTAGTTACTCAACTGG	M-2	(663+38)
M-R1027	CAGGAAACAGCTATGACCAGTAGAAACAAGGTAGTTTTT		
NS-F0	TGTAAAACGACGGCCAGTAGCAAAAGCAGGGTG	NS-1	(630+38)
NS-R607	CAGGAAACAGCTATGACCAAGCGAATCTCTG		
NS-F439	TGTAAAACGACGGCCAGTCTTAGAGCTTTTAC	NS-2	(422+38)
NS-R860	CAGGAAACAGCTATGACCAGTAGAAACAAGGGTGTTTT		

Table S3 The available full-length sequences of selected influenza A (H7N9) viruses

Viral Strains	Collection Date	GISAID Isolate ID
A/Shanghai/1/2013	Feb-2013	EPI_ISL_138737
A/Anhui/1/2013	Mar-2013	EPI_ISL_138739
A/Zhejiang/1/2013	Mar-2013	EPI_ISL_139652
A/Jiangsu/1/2013	Mar-2013	EPI_ISL_146437
A/Fujian/01/2013	Apr-2013	EPI_ISL_141180
A/Fujian/05/2013	Apr-2013	EPI_ISL_157292
A/Fujian/02/2013	Apr-2013	EPI_ISL_157288
A/Chicken/Shanghai/S1053/2013	Apr-2013	EPI_ISL_138983
A/Environment/Shanghai/S1088/2013	Apr-2013	EPI_ISL_138984
A/environment/Fujian/SC337/2013	Apr-2013	EPI_ISL_142914
A/Beijing/02/2013	May-2013	EPI_ISL_157286
A/Taiwan/3/2013	Dec-2013	EPI_ISL_166324
A/Shanghai/01/2014	Jan-2014	EPI_ISL_162470
A/Fujian/1/2014	Jan-2014	EPI_ISL_192451
A/Fujian/5/2014	Jan-2014	EPI_ISL_192411
A/silky_chicken/Hong_Kong/1772-3/2014	Jan-2014	EPI_ISL_155815
A/Environment/Hunan/07836/2014	Jan-2014	EPI_ISL_192437
A/Zhejiang/21/2014	Feb-2014	EPI_ISL_192345
A/Fujian/15/2014	Feb-2014	EPI_ISL_192392
A/Hong Kong/8113530/2014	Mar-2014	EPI_ISL_157703
A/Taiwan/1/2014	Apr-2014	EPI_ISL_160435
A/Xinjiang/98692/2014	Nov-2014	EPI_ISL_172826
A/Fujian/17/2014	Nov-2014	EPI_ISL_192336
A/Fujian/19/2014	Dec-2014	EPI_ISL_192337
A/Environment/Fujian/23611/2014	Dec-2014	EPI_ISL_192476
A/Fujian/6/2015	Jan-2015	EPI_ISL_192281
A/Hong Kong/2550/2015	Jan-2015	EPI_ISL_170981
A/British Columbia/1/2015	Jan-2015	EPI_ISL_171342
A/Guangdong/15SF001/2015	Jan-2015	EPI_ISL_192310
A/Zhejiang/1/2015	Jan-2015	EPI_ISL_192293
A/Fujian/10/2015	Jan-2015	EPI_ISL_223670
A/Fujian/16/2015	Jan-2015	EPI_ISL_223672
A/Fujian/18/2015	Jan-2015	EPI_ISL_223673
A/Fujian/22/2015	Jan-2015	EPI_ISL_223680
A/Chicken/Guangdong/HZ098/2015	Jan-2015	EPI_ISL_176824
A/Fujian/27/2015	Feb-2015	EPI_ISL_192502
A/Anhui/33224/2015	Mar-2015	EPI_ISL_192494
A/Fujian/1/2016	Jan-2016	EPI_ISL_233628
A/Fujian/3/2016	Jan-2016	EPI_ISL_233629

A/Hong Kong/VB16021618/2016	Feb-2016	EPI_ISL_212471
A/Hong Kong/VB16049808/2016	Mar-2016	EPI_ISL_215688
